# Higher disease burden and greater small fibre impairment in women with painful diabetic neuropathy

**DOI:** 10.1097/PR9.0000000000001443

**Published:** 2026-04-28

**Authors:** Gordon Sloan, Dinesh Selvarajah, Jessica Smith, Shillo Pallai, Marni Greig, M. Shaan Goonoo, Thomas Cash, Merhawit A. Abera, Sharon Caunt, Iain D. Wilkinson, Solomon Tesfaye

**Affiliations:** a Division of Clinical Medicine, University of Sheffield, Sheffield, United Kingdom; b Diabetes Research Unit, Sheffield Teaching Hospitals NHS Foundation Trust, Sheffield, United Kingdom; c Leighton Hospital, Mid Cheshire Hospitals NHS Foundation Trust, Crewe, England, United Kingdom; d Mekelle University, Mek'ele, Tigray, Ethiopia

**Keywords:** Neuropathy, Neuropathic pain, Painful diabetic neuropathy, Diabetes

## Abstract

**Objective:**

Although the incidence of Painful-Diabetic Peripheral Neuropathy (Painful-DPN) is higher in women, the underlying reasons for this are not known. This study investigated sex differences in pain and neurological phenotypes in 2 cohorts of patients with Painful-DPN.

**Methods:**

Two cohort analyses were performed in this retrospective analysis of participants with Painful-DPN recruited to cross-sectional studies: (1) Patient-Reported Outcomes Measures Cohort analysis (n = 184), which assessed neuropathic pain intensity (Numeric Rating Score), neuropathic symptoms (Neuropathic Pain Scale), mood (Hospital Anxiety and Depression Scale), quality of life (Norfolk QoL-DN), and sleep (Medical Outcomes Sleep Study); (2) Neurological Phenotyping Cohort analysis (n = 100), which assessed large- (Neuropathy Impairment Score of the Lower Limb plus Seven Neurophysiological Tests) and small-nerve fibre function. Participants were also divided into irritable (IR) and nonirritable nociceptor phenotypes.

**Results:**

In the first cohort analysis, women reported greater neuropathic pain symptom burden, poorer sleep, and higher anxiety prevalence than men. In the second cohort analysis, men had greater large-nerve fibre impairment, whereas women exhibited more severe small-fibre dysfunction. In addition, IR nociceptor phenotypes were more prevalent in women.

**Conclusion:**

Women with Painful-DPN experience a greater disease burden, increased small-fibre impairment, and are more likely to have the IR nociceptor phenotype, suggesting potential sex-specific disease mechanisms.

## Abstract 2

Supplemental Digital Content is Available in the Text.

In this 2-cohort analysis, women with painful diabetic neuropathy have higher pain, anxiety, sleep disturbance, and worse small-fibre dysfunction compared with men.

## 1. Introduction

Painful-Diabetic Peripheral Neuropathy (Painful-DPN) is one of the commonest causes of chronic pain globally, occurring in up to a third of people with diabetes.^35^ It presents with a range of unpleasant symptoms, including burning, electric shock-like pains, allodynia, and hyperalgesia, resulting in at least moderate to severe pain in over 70% of cases.^18^ Unsurprisingly, Painful-DPN has a profound impact on its sufferers' lives, leading to mood disorders,^33^ sleep impairment, and a reduced quality of life.^18^ Unfortunately, the current treatments for Painful-DPN are only partially effective at best,^42^ in large part, this is because of a lack of understanding of the disease mechanisms underlying Painful-DPN.^35^ One notable knowledge gap is the lack of study into sex differences.

Cross-sectional studies have reported a greater prevalence of Painful-DPN in women compared with men.^1,21,44^ We also recently published data from the EURODIAB Prospective Complications Study and found that female sex was a risk factor for Painful-DPN,^15^ a finding confirmed in other longitudinal studies since.^6,22^ These findings suggest that disease burden and underlying mechanisms may differ between men and women with Painful-DPN; however, these studies did not include detailed neurophysiological, psychometric, and quality of life assessments. Thus, in this study, we performed a retrospective analysis to examine if pain and neurological phenotypes are different between women and men in 2 cohorts of patients with Painful-DPN.

## 2. Research design and methods

### 2.1. Study design and participants

The study is a retrospective analysis of all research participants with Painful-DPN (n = 316) who were recruited into cross-sectional studies between 2009 and October 2024 at Sheffield Teaching Hospitals NHS Foundation Trust.^33,34^ We included all participants fulfilling the inclusion/exclusion criteria, 184 of whom underwent detailed psychometric assessments of pain intensity, mood, sleep, and quality of life (Patient Reported Outcome Measures [PROM] Cohort Analysis) and formed the first cohort. One hundred participants had detailed neurophysiological tests (Neurological Phenotyping Cohort Analysis) and formed the second cohort.

All participants were recruited from outpatient diabetes clinics at Sheffield Teaching Hospitals NHS Foundation Trust (United Kingdom), aged 18 to 85 years diagnosed with diabetes >6 months previous and symptoms of Painful-DPN for at least 6 months. Exclusion criteria included pregnancy, insufficient command of the English language to complete questionnaires, concurrent severe psychiatric conditions, history of substance abuse including excess alcohol consumption (>20 units per week), at least moderate pain from causes other than Painful-DPN, neuropathies not caused by diabetes, or other diabetic neuropathies. All participants recruited to studies gave informed consent before participating in data collection. The individuals included were participating in studies that had prior ethics approval by Research Ethics Committees.^33,34^

### 2.2. Case definition and participant assessments

#### 2.2.1. Patient-reported outcome measures analysis

Probable DPN was diagnosed based on the presence of a combination of symptoms and signs of neuropathy including any 2 or more of neuropathic symptoms, decreased distal sensation, or unequivocally decreased or absent ankle reflexes, according to the Toronto criteria.^40^ Painful-DPN was defined according to the IASP definition of “Probable neuropathic pain,” ie, pain associated with sensory signs in the same neuroanatomically plausible distribution.^17^

Participants underwent detailed clinical history, physical examination, blood testing, and questionnaire-based assessment of mood, sleep, quality of life, and neuropathic pain characteristics. The severity of neuropathic pain over the last 24 hours was assessed using an 11-point numeric rating scale (NRS) ranging from 0 (no pain) to 10 (most pain imaginable).

The validated questionnaires participants underwent included the following:

Hospital anxiety and depression scale (HADS): This questionnaire is a measure of anxiety and depression.^48^ Anxiety and depression subscales are assessed in 7 items on scales ranging from no feelings of anxiety or depression (score 0) to severe feelings of anxiety or depression (score 3). Scores range from 0 to 21 in each subscale (0–7 normal, 8–10 borderline, 11–14 abnormal, 15–21 severe).

Medical outcomes study sleep scale (MOS Sleep): This questionnaire is a 12-item assessment of dimensions of sleep quality over the preceding 4 weeks, including initiation, maintenance, respiratory problems, quantity, perceived adequacy, and somnolence.^31^ Individual scores of the 12 items can be combined to calculate 2 summary indices: sleep index 1, summarises sleep disturbances, sleep adequacy, respiratory impairment, and somnolence domains; sleep index 2, summarises all of the points in index 1 and also additional measures of sleep disturbance (time taken to fall asleep, perception that sleep was not quiet) and somnolence (perception of how often the person feels drowsy during the day). Higher scores of these indices suggest worse sleep quality.

Norfolk quality of life-DN: This is a DPN-specific questionnaire used to assess quality of life.^45^ The questionnaire assesses 4 subscales of quality of life related to DPN (activities of daily living, small-fibre, large-fibre, and autonomic fibre-related disability) and 1 summary measure of generic health status with higher values suggestive of greater impairment.

Neuropathic pain scale: This questionnaire is a 10-question instrument, which assesses the quality of neuropathic pain, including intensity, heat, dullness, sharpness, sensitivity, and unpleasantness.^19^ Each measure is assessed using an 11-point NRS ranging from 0 (no pain) to 10 (most pain imaginable). These subscales are combined to form a total score, with higher levels suggestive of greater intensity of neuropathic symptoms.

Brief pain inventory short form for diabetic peripheral neuropathy (BPI-DPN): This questionnaire evaluates the psychometric properties of the modified Brief Pain Inventory for patients with Painful-DPN.^46^ It consists of a 4-item pain severity scale, a 7-item pain interference scale, and questions assessing the location of pain and efficacy of pain medications. Pain severity scores and pain interference scales are measured on an 11-point NRS, with 0 representing a low score (ie, no pain or pain not interfering) and 10 representing a high score (ie, pain as bad as you can imagine, or pain completely interferes).

Pain catastrophizing scale: This questionnaire evaluates the degree to which participants have certain thoughts and feelings when experiencing pain using a Likert scale ranging from 0 (not at all) to 4 (all the time).^39^ Summary scores are calculated for 3 domains of catastrophizing (rumination, magnification, and helplessness).

Chronic pain acceptance questionnaire: This questionnaire assesses the ability of participants to adapt to chronic pain and generates 2 summary subscores for “activities engagement” and “pain willingness.”^26^ Higher scores in activities engagement suggest that patients are more likely to pursue activities despite the pain. Higher scores in pain willingness indicate a recognition that avoidance and control are often unworkable methods of adapting to chronic pain.

#### 2.2.2. Neurological phenotyping cohort analysis

Confirmed DPN was diagnosed according to the American Academy of Neurology minimum case definition criterion (ie, an abnormality [>99th or <first percentile]) of any attribute of nerve conduction in 2 separate nerves, one of which must be the sural nerve.^16^ Painful-DPN was confirmed according to the IASP definition of “Definite neuropathic pain” (diagnostic test confirming a lesion or disease of the somatosensory nervous system explaining the pain),^17^ and a Douleur Neuropathique 4 (DN4) score ≥4.^5^

Participants underwent detailed clinical history, physical examination, and detailed neurophysiological testing, by members of the research team using the same equipment and receiving the same training for each test.

Assessment of peripheral neurological status was determined clinically using 2 clinical scoring systems: (1) Toronto Clinical Neuropathy Score (TCNS)^8^ and (2) Neuropathy Impairment Score of the Lower Limb (NIS-LL).^7^

Nerve conduction studies were performed at a stable skin temperature of 31°C and a room temperature of 24°C using a Medelec electrophysiological system (Synergy Oxford Instruments, Oxford, United Kingdom). The following nerve attributes were measured: (1) sural sensory nerve action potentials and conduction velocities; (2) common peroneal distal latency, compound muscle action potential, and conduction velocity; and (3) tibial motor nerve distal latency. In addition, Cardiac Autonomic Function was performed using the O'Brien protocol,^30^ and vibration detection thresholds were acquired from the dorsal aspect of the right foot using the Computer-Assisted Sensory Evaluation IV (WR Electronics, Stillwater, MN) using the 4-, 2-, and 1-step algorithm.^12^ A neuropathy composite score was calculated by combining the NIS-LL with 7 neurophysiological tests (cardiac autonomic function; sural sensory nerve action potentials; peroneal nerve action potential, conduction velocity, and distal latency; tibial nerve distal latency; and vibration detection thresholds (VDT) using the 4-, 2-, 1-step algorithm) to determine the NIS(LL) plus 7 neurophysiological tests score (NIS[LL]+7),^11^ continuous measure of neuropathy severity.

Quantitative sensory testing (QST) was also performed according to the German Research Network on Neuropathic Pain (DFNS) protocol.^32^ Using this standardised protocol, the following QST measures were obtained: cold and warm detection thresholds (WDT) and thermal sensory limen (TSL); paradoxical heat sensations; mechanical detection thresholds (MDT) and VDT; mechanical pain threshold, dynamic mechanical allodynia, mechanical pain sensitivity, and pressure pain threshold; and wind-up ratio. The QST data were entered into the data analysis system eQUISTA provided by the DFNS. eQUISTA transformed the raw QST data into *z* scores thus normalising for age, sex, and the body location of testing. Positive *z* scores denote gain of function, whereas negative *z* scores denote loss of function; abnormal results are defined as z > 1.96 or < −1.96.

On the basis of QST results, participants were subgrouped into 1 of 2 phenotypes according to methods described previously^34^: (1) irritable nociceptor phenotype (IR), including the presence of dynamic mechanical allodynia, reduced mechanical pain threshold or PPT, increased mechanical pain sensitivity, lower CPT or HPT, or any combination of these signs of neuronal hyperexcitability, irrespective of other QST results; (2) nonirritable nociceptor phenotype, including sensory loss with none of the signs of hyperexcitability within the IR group.

## 3. Statistical analysis

Analysis was performed using the statistical package Statistical Product and Service Solutions Version 26 (SPSS, IBM Corporation, New York). Data were tested for normality using the Shapiro–Wilk test. Normally distributed characteristics are presented as means and standard deviations, and those with a nonparametric distribution are presented as medians and interquartile ranges. Categorical and dichotomous variables are presented as number of cases and group percentages. Group differences between women and men were compared using the independent-samples *t* test for normally distributed continuous data (with the 95% confidence interval of the difference) and the Mann–Whitney *U* test for non-normally distributed data. χ^2^ test was used to compare categorical and dichotomous variables. The Spearman correlation test was used to measure the association between pain intensity (NRS) and psychometric and neurological phenotyping variables; these analyses were done within the whole cohort and then divided by sex.

No prior sample size calculation was performed, as this is an exploratory study using previously acquired data from completed cross-sectional studies.

The data sets generated during the current study are available from the corresponding author upon reasonable request.

## 4. Results

### 4.1. Patient-reported outcome measures analysis

A total of 184 participants, 69 women and 115 men, underwent PROM assessments, displayed in Table 1.

**Table 1:** Demographic and clinical characteristics and questionnaire results of participants undergoing analysis of patient reported outcome measures grouped by sex.

	Women (n = 69)	Men (n = 115)	*P*
Clinical and demographic variables			
Age, y	60.0 (16.0)	63.0 (16.0)	0.147
Duration of diabetes, y*	18.0 (13.0)	16.0 (16.0)	0.501
HbA1c, mmol/mol*	68.3 (22.8)	68.3 (24.9)	0.839
Type of diabetes, n (%)*			**0.008** †
Type 1	20 (29%)	15 (13%)	
Type 2	49 (71%)	100 (87%)	
Body mass index, kg/m^2^*	32.7 ± 6.9	31.9 ± 5.8	0.226‡*
Pain intensity, numeric rating scale	7.0 ± 2.2	6.4 ± 2.5	0.045‡*
Duration of pain, y*	8.0 (6.0)	7.0 (6.0)	0.452
Type of analgesic medication prescribed*			
Tricyclic antidepressants, %	30.2%	28.7%	0.504
Serotonin noradrenaline reuptake inhibitors, %	7.5%	13.8%	0.268
Anticonvulsants, %	34.0%	36.3%	0.787
Opioids, %	23.1%	16.5%	0.345
Neuropathic pain scale (NPS)*			
Total score	56.3 ± 18.3	49.8 ± 19.3	**0.021**
Brief pain inventory-DPN pain intensity scores*			
Worst pain	8.0 (2.8)	7.0 (3.0)	**0.021**
Average pain	5.0 (4.0)	5.0 (3.0)	0.058
Least pain	2.0 (4.0)	2.0 (4.0)	0.945
Pain now	3.0 (6.0)	3.0 (4.0)	0.930
Brief pain inventory-DPN pain interference scores*			
General activity	5.0 (7.0)	5.0 (6.0)	0.858
Mood	5.0 (6.8)	4.0 (6.0)	0.313
Walking ability	6.0 (5.0)	7.0 (6.0)	0.261
Normal work	4.0 (7.0)	5.0 (8.0)	0.467
Relations with other people	3.5 (6.0)	2.0 (5.0)	0.659
Sleep	7.0 (5.0)	5.0 (7.0)	**0.017**
Enjoyment of life	5.0 (6.0)	5.0 (6.0)	0.608
Norfolk quality of life-DN*			
Large fibre	13.3 ± 8.0	12.9 ± 6.8	0.363
Small fibre	8.0 (6.0)	5.0 (7.0)	**0.031**
Autonomic	4.0 (6.0)	5.0 (4.0)	0.071
Activities of daily living	6.0 (10.0)	5.0 (9.0)	0.489
Total score	30.5 (25.0)	31.0 (28.0)	0.619
Medical outcomes study sleep scale*			
Sleep disturbance	59.0 (52.9)	32.8 (47.5)	0.126
Somnolence	40.0 (40.0)	40.0 (35.0)	0.620
Sleep adequacy	40.0 (42.5)	40.0 (40.0)	0.875
Snoring	20.0 (60)	40.0 (57.0)	0.441
Awaken short of breath or with headache	20.0 (60.0)	0.0 (20.0)	**0.017**
Quantity of sleep, h	6.0 (2.0)	7.0 (3.0)	0.183
Time to fall asleep, h	2.5 (4.0)	2.0 (3.0)	0.376
Index 1	43.8 ± 21.2	35.3 ± 21.7	**0.009** ‡
Index 2	47.6 ± 21.9	39.8 ± 22.0	**0.027** ‡
Hospital anxiety and depression scale*			
Anxiety score	7.0 (9.0)	6.0 (6.0)	**0.022**
Anxiety diagnosis, n (%)	18 (37.5%)	17 (21.8%)	**0.028** †
Depression score	6.0 (6.0)	6.0 (6.0)	0.866
Depression diagnosis, n (%)	9 (18.8%)	23 (29.5%)	0.145

Data are presented as mean (±SD), median (IQR), or number (%).

Boldface text denotes significant results.

* Missing data: duration of diabetes, n = 4; HbA1c, n = 26; body mass index, n = 13; duration of pain, n = 52; analgesia prescription, n = 69; neuropathic pain scale, n = 32; BPI DPN pain intensity, n = 53; Norfolk QoL, n = 47; MOS sleep, n = 34; HADS, n = 58. Tests were Mann–Whitney U, unless otherwise stated.

† Chi^2^ test.

‡ Independent *t* test.

BPI-DPN, brief pain inventory; diabetic peripheral neuropathy; HADS, hospital anxiety and depression scale.

The median (IQR) age of participants was 60.1 (17.0) years, the duration of diabetes was 16.0 (14.0) years, and the HbA1c was 68.3 mmol/mol (22.2). The mean body mass index (±SD) of participants was 32.7 (±6.3) kg/m^2^. The ethnicity of the population was White British except for 5 individuals who were Afro-Caribbean (3%). There was a greater proportion of type 1 diabetes and lower proportion of type 2 diabetes in women compared with men (Chi^2^ test *P* = 0.008).

**Figure 1.**
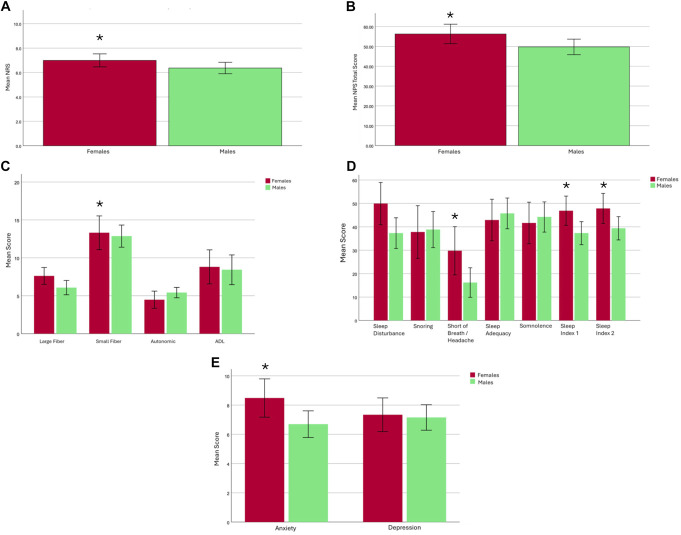
Patient-reported outcome measures cohort analyses between women (red) and men (green). (A) Mean Numeric Rating Scale (NRS) for pain over 24 hours; (B) Mean Neuropathic Pain Scale (NPS) Total Score; (C) Mean Score for Norfolk Quality of Life Diabetic Neuropathy domains; (D) Mean Score for Medical Outcomes Study Sleep Scale subscores and Sleep Index 1 and 2; (E) Mean Score of Hospital Anxiety and Depression Scale for subscores of Anxiety and Depression. Data presented as mean ± 95% confidence interval. Data highlighted with “*” signify group difference between men and women.

The severity of NRS was significantly greater in women (mean ± SD, 7.0 ± 2.2) compared with men (6.4 ± 2.5, independent *t* test, *P* = 0.045, 95% CI −1.35, 0.10), Figure 1A. The NPS total score was significantly higher in women (56.3 ± 18.3) than men (49.8 ± 19.3, independent *t* test *P* = 0.021), Figure 1B. There were no significant differences in NPS subscores, other than itch, which was significantly higher for women (median [IQR], 2.0 [7.0]) than men (0.0 [4.0], Mann–Whitney *U* test, *P* = 0.034; see supplemental digital content, Materials, http://links.lww.com/PR9/A406).

The median (IQR) worst pain score in the BPI-DPN was higher in women (8.0 [2.8]) compared with men (7.0 [3.0] Mann–Whitney *U* test, *P* = 0.021), Table 2, although there was missing data in 53 individuals. Within the BPI-DPN measures of pain interference, the sleep score was significantly higher in women (7.0 [5.0]) compared with men (5.0 [7.0], *P* = 0.021), with no other differences in other measures. There were no differences in the total score of the Norfolk Quality of Life-DN, but the small-fibre subscore was significantly higher in women (8.0 [6.0]) vs men (5.0 [7.0], *P* = 0.031; Fig. 1C).

**Table 2:** Demographic, clinical, and neurological phenotyping characteristics of participants within the neurological phenotyping cohort analysis grouped by sex.

	Women (n = 38)	Men (n = 62)	*P*
Clinical and demographic variables			
Age, y	59.0 (16.0)	59.5 (13.0)	0.904
Duration of diabetes, y*	11.5 (17.0)	13.0 (17.0)	0.358
HbA1c, mmol/mol*	64.0 (29.8)	66.0 (22.0)	0.900
Type of diabetes*			0.803†
Type 1	9 (27%)	14 (24%)	
Type 2	25 (73%)	44 (76%)	
Body mass index, kg/m^2^	31.0 ± 6.6	30.9 ± 6.3	0.478‡
Systolic blood pressure, mm Hg	136.3 ± 16.8	134.5 ± 19.4	0.325‡
Clinical scoring measures of neuropathy			
NIS-LL	15.7 ± 15.1	20.0 ± 11.5	0.073‡
TCNS*	10.8 ± 3.2	13.7 ± 3.8	**0.002** ‡
Neuropathy composite score			
NIS-LL+7	22.8 ± 16.6	29.1 ± 14.5	**0.024** ‡
Nerve conduction study variables*§			
Sural nerve amplitude	5.50 (9.61)	0.54 (7.40)	**0.027**
Sural nerve velocity	37.05 (7.85)	34.90 (11.90)	0.400
Peroneal nerve amplitude	2.40 (2.85)	1.50 (3.53)	0.233
Peroneal nerve velocity	38.35 (7.23)	33.00 (39.30)	**0.033**
Peroneal nerve latency	5.40 (2.00)	5.75 (2.70)	0.454
Tibial nerve latency	5.16 (1.97)	5.60 (4.25)	0.513
Quantitative sensory testing			
CDT	−2.29 ± 1.34	−2.24 ± 0.96	0.413‡
CDT abnormal, n (%)	23 (64%)	27 (56%)	0.480†
WDT	−2.07 ± 0.92	−1.60 ± 0.55	**0.003** ‡
WDT abnormal, n (%)	25 (69%)	14 (29%)	**<0.001** †
TSL	−2.66 ± 1.07	−1.78 ± 0.63	**<0.001** ‡
TSL abnormal, n (%)	28 (78%)	19 (40%)	**<0.001** †
CPT	−0.65 ± 0.90	−0.63 ± 0.78	0.457‡
CPT abnormal, n (%)	0 (0%)	1 (2%)	0.384†
HPT	−1.29 ± 1.53	−1.07 ± 1.01	0.213‡
HPT abnormal, n (%)	13 (36%)	2 (4%)	**<0.001** †
PPT	1.40 ± 2.10	0.65 ± 1.82	**0.046** ‡
PPT abnormal, n (%)	17 (47%)	15 (31%)	0.136†
VDT	−1.59 ± 2.25	−3.53 ± 2.51	**<0.001** ‡
VDT abnormal, n (%)	14 (39%)	33 (69%)	0.006†
MPT	−1.60 ± 1.81	−1.39 ± 1.64	0.306‡
MPT abnormal, n (%)	23 (63%)	27 (57%)	0.619†
MPS	1.39 ± 1.81	−1.12 ± 1.64	0.140‡
MPS abnormal, n (%)	17 (47%)	23 (48%)	0.941†
MDT	−2.35 ± 1.95	−2.66 ± 1.89	0.495‡
MDT abnormal, n (%)	19 (53%)	29 (61%)	0.473†
WUR abnormal, n (%)	0 (0%)	1 (2%)	0.367
PHS abnormal, n (%)	15 (42%)	16 (33%)	0.477†
DMA abnormal, n (%)	6 (16%)	3 (6%)	0.217†
Irritable nociceptor phenotype	20 (56%)	15 (31%)	**0.027** †

Data are presented as mean (±SD), median (IQR), or number (%).

Boldface text denotes significant results.

* Missing data: duration of diabetes, n = 4; HbA1c, n = 19; type of diabetes, n = 8; systolic blood pressure, n = 18; TCNS, n = 41; quantitative sensory testing, n = 19.

† Chi^2^ test.

‡ Unable to obtain/detect NCS parameters: sural nerve amplitude, n = 15; sural nerve velocity, n = 51; peroneal nerve amplitude, n = 14; peroneal nerve velocity, n = 13; peroneal nerve latency, n = 11; tibial nerve latency, n = 5; QST, n = 16. All tests were Mann–Whitney U, unless otherwise stated.

§ Independent *t* test.

NIS-LL, neuropathy impairment score of the lower limbs; TCNS, Toronto clinical neuropathy score; NIS-LL+7, neuropathy impairment score of the lower limbs plus 7 neurophysiological tests; CDT, cold detection threshold; WDT, warm detection threshold; TSL, thermal sensory limen; CPT, cold pain threshold; HPT, heat pain threshold; PPT, pressure pain threshold; VDT, vibration detection threshold; MPT, mechanical pain threshold; MPS, mechanical pain sensitivity; MDT, mechanical detection threshold; WUR, wind-up ratio; PHS, paradoxical heat sensation; DMA, dynamic mechanical allodynia.

Both sleep indices of the MOS sleep were higher in women (mean ± SD: sleep index 1, 43.8 ± 21.2; sleep index 2, 47.6 ± 21.9) compared with men (sleep index 1, 35.3 ± 21.7, independent *t* test, *P* = 0.009; sleep index 2, 39.8 ± 22.0, *P* = 0.027; Fig. 1D). The HADS subscore for anxiety was significantly higher in women (median [IQR], 7.0 [9.0]) than men (6.0 [6.0], Mann–Whitney *U* test, *P* = 0.022), and there was also a greater proportion of women with anxiety (n = 18 [37.5%]) compared with men (n = 17 [21.8%], Chi^2^ test *P* = 0.028; Fig. 1E). Whereas there was no difference in depression between sexes were also no differences in measures within the PCS nor the CPAQ (see supplemental digital content, Materials, http://links.lww.com/PR9/A406).

Correlation analysis showed pain severity correlated with HADS anxiety (*r* 0.340, *P* < 0.001) and depression (*r* 0.273, *P* < 0.001) subscales, MOS sleep indices 1 and 2 (sleep index 2: *r* 0.405, *P* < 0.001), and the Norfolk Quality of Life Total Score (*r* 0.405, *P* < 0.001). These correlations remain significant when the total cohort was subgrouped into men or women.

### 4.2. Neurological phenotyping cohort analysis

A total of 100 participants, 38 women and 62 men, underwent detailed neurological phenotyping.

The demographic, clinical measures, and neurological phenotyping results of participants involved in this cohort analysis are displayed in Table 2. The median (IQR) age of participants was 59.0 (14.0) years, duration of diabetes was 12.5 (17.0) years, and the HbA1c was 65.0 (23.0) mmol/mol. The mean body mass index (±SD) was 30.9 (±6.4) kg/m^2^. The ethnicity of the population was 94% White British except with 2% British African-Caribbean, and 1% for British Asian, British Indian, Jewish, and Mixed each. There were no differences between women and men in these demographic and clinical measures. In this smaller cohort than the PROM analysis, the median intensity of neuropathic pain NRS was numerically higher in women (Median 7.0 [IQR 3.0]) than men (6.0 [4.0]), although there was no statistical difference (Mann–Whitney *U* Test *P* = 0.437).

The TCNS was significantly higher in men (mean ± SD, 13.7 ± 3.8) compared with women (10.8 ± 3.2, independent *t* test *P* = 0.002). There was a trend toward the NIS-LL being greater in men than women (*P* = 0.073). However, the neuropathy composite score combining the NIS-LL with 7 neurophysiological tests was significantly higher in men (29.1 ± 14.5) compared with women (22.8 ± 16.6, *P* = 0.024). Moreover, all neurophysiological tests showed numerically more severe neuropathy in men than women, with group differences reaching significance in sural nerve amplitude (Mann–Whitney U, *P* = 0.027) and peroneal nerve velocity (Mann–Whitney U, *P* = 0.033).

**Figure 2.**
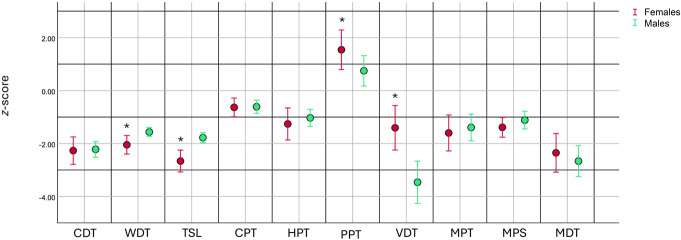
Sex-specific somatosensory profiles of participants in the neurological phenotyping cohort analysis. Mean of *z*-values and 95% confidence interval. CDT, cold detection threshold; WDT, warm detection threshold; TSL, thermal sensory limen; CPT, cold pain threshold; HPT, heat pain threshold; PPT, pressure pain threshold; VDT, vibration detection threshold; MPT, mechanical pain threshold; MPS, mechanical pain sensitivity; MDT, mechanical detection threshold. Data presented as mean and 95% confidence interval. Data highlighted with “*” signify group difference between men and women.

The VDT, a measure of large-fibre function, showed a loss of function in men (−3.53 ± 2.51) compared with women (−1.59 ± 2.25, independent *t* test *P* < 0.001), shown in Figure 2. Whereas measures of small-fibre function, WDT and TSL, were lower, suggesting a greater loss of function in women compared with men (*P* = 0.003 and *P* ≤ 0.001, respectively). Moreover, the PPT was higher in women (1.40 ± 2.10) compared with men (0.65 ± 1.82, independent *t* test *P* = 0.046), suggesting a gain of function in the former (Fig. 2). There was further evidence of a gain of function in women compared with men, with more women (n = 20 [56%]) being classified as having the IR nociceptor phenotype than men (n = 15 [31%], Chi^2^ test *P* = 0.027).

Correlation analysis was performed between individual measures of DFNS QST and also the NIS-LL+7 and pain intensity in women and men. The NIS-LL+7 (Spearman r, *P*-value; 0.360, *P* = 0.008), VDT (r −0.460, *P* = 0.002), and MDT (r −0.526, *P* < 0.001) correlated with pain intensity in men but not women. In women, however, PPT correlated with neuropathic pain intensity (r −0.427, *P* = 0.018) but not men.

## 5. Discussion

The main findings of the study based on analysis of 2 separate cohorts were that pain severity and small-fibre impairment was greater in women compared with men. On assessment of PROM outcomes, there was a greater burden of disease in women as shown by higher pain intensity, symptoms score (NPS) and itching subscore, “worst-pain” score on BPI-DPN, small-fibre domain on Norfolk-QoL DN, anxiety, and worse sleep outcomes. Pain intensity correlated with anxiety, depression, sleep quality, and quality of life measured by the Norfolk QoL-DN. The Neurological Phenotyping Cohort Analysis showed greater large-fibre dysfunction in men compared with women. However, measures of small-fibre function, WDT and TSL, were more impaired in women compared with men, which is a novel finding. Moreover, women had gain of function of PPT, and a greater proportion of women with the IR phenotype compared with men. Finally, measures of large-fibre impairment correlated with pain intensity in men, but not women.

Experimental pain studies have shown higher sensitivity to pain in women compared with men.^24,29^ Our finding of greater pain intensity in women with Painful-DPN compared with men is consistent with other recent studies.^2,9^ There were some measures of pain intensity, which trended towards greater pain in women but did not reach significance, as a result of missing data (BPI-DPN average pain) or lower sample size (Neurological Phenotyping Cohort Analysis); however, pain intensity always trended towards being greater in women than men. We also demonstrate that “worst pain” on BPI-DPN is higher in women, with a trend towards several neuropathic symptoms on NPS being more intense in women compared with men (eg, sharp, hot, dull, cold, etc.) and “itch” reaching statistical significance, a finding that aligns with evidence from the literature.^38^ These findings are of interest, and future larger prospective studies with adequate sample sizes should aim to characterize the clinical pain phenotypes of Painful-DPN in women and men.

There is a well-recognised association between Painful-DPN and mood disorders and sleep impairment.^20,33^ Indeed, greater intensity of neuropathic pain corresponds with higher levels of anxiety and insomnia.^20^ In addition, at a population level, women are at greater risk of developing anxiety^3^ and sleep disorders.^25^ Thus, our findings of greater levels of anxiety and sleep impairment in women with Painful-DPN are consistent with the literature. The neurobiological mechanisms underlying the sex-specific associations between pain, anxiety, and sleep remain incompletely understood, with a potential bidirectional relationship. Studies suggest that experimental sleep disruption may influence pain perception through distinct sex-specific pathways, with women exhibiting a decreased heat pain threshold and impaired descending pain inhibition.^14,36^ Anxiety in women may contribute to amplification of pain perception, leading to lower pain thresholds, and diminishing analgesic effectiveness.^13,47^ As our study was cross-sectional and did not include a control group, causality cannot be inferred, but these findings provide further evidence that the burden of Painful-DPN is greater in women compared with men.

Our study is unique in its detailed comparison of neurological phenotypes between men and women by using both the DFNS Quantitative Sensory Testing (QST) protocol and conventional nerve conduction studies. The finding that there are different phenotypes of Painful-DPN between men and women is consistent with preclinical and clinical data.^27^ Preclinical studies have demonstrated greater mechanical allodynia in female rodents^10,23^ and also greater nerve conduction abnormalities in male rodents.^10^ In clinical studies, the EURODIAB Prospective Complications Study found that women with Painful-DPN had a lower level of diabetic nephropathy suggesting women may develop neuropathic pain at an earlier stage compared with men.^41^ Recent data from the DCCT/EDIC cohort study also found that women were more at risk of developing Painful-DPN without clinical signs.^6^ However, both these large prospective studies did not use validated assessments for neuropathic pain and did not measure small-fibre function. Another recent study that included 2 cohorts, one with a broad spectrum of nerve injury and another with DPN only, found that women experienced more intense pain despite milder nerve impairment on electrophysiological studies compared with men.^2^ In contrast to our findings, however, the study reported less advanced small-fibre dysfunction in women compared with men, using Laser Doppler Imaging flare and Cooling Detection Thresholds. Another large cohort study using DFNS QST to a large number of people with polyneuropathy (n = 571) of various aetiologies, including 84 with Painful-DPN, found that MDT was lower in men compared with women with no other sex differences in other sensory domains.^28^ Although this was a large study, the Painful-DPN group was not examined independently. Thus, ours is the only study to use the DFNS QST to directly compare men and women in a well-characterized Painful-DPN cohort.

Although women demonstrated greater loss in thermal detection (WDT and TSL), a higher proportion were also classified as having the IR phenotype. This likely reflects greater small-fibre dysfunction in women with coexistent gain-of-function; however, in this study, direct examination of small-nerve fibres (eg, with skin biopsy) was not performed and would be recommended in future studies. The Pain in Neuropathy Study (PiNS) showed that pain intensity correlated with neuropathy severity in Painful-DPN assessed using TCNS and greater proximal spread of clinical signs.^43^ In our study, we also demonstrate that pain intensity correlates with large-fibre dysfunction but only in men, indicating the presence of sex-specific differences in the neuropathy phenotype of Painful-DPN. Our study also found a higher small fiber neuropathy score in the Norfolk QOL-DN; however, this subscore is less specific to small fiber function than the detailed DFNS QST measures.

Our study suggests that women with Painful-DPN are suffering with a greater disease burden than men and that also there may be distinct sensory profiles, which differentiate the sexes. Although recent studies have confirmed there are sex differences in Painful-DPN,^6,9,15^ significant gaps remain in our understanding of the underlying mechanisms. In addition to biological factors, psychological and sociocultural factors such as stereotypes/expectations, gender identity, and behavioural/emotional responses to pain are probably involved.^24^ Neurobiological factors implicated in sex-specific mechanisms in pain include alterations in pain processing in the brain, neuropeptide functioning, hormone levels, and different innate and adaptive immune system responses to neuropathic pain.^24,29^ Moreover, recent data have demonstrated sexual dimorphism of nociceptor function.^4,37^ Our findings have potential implications for clinical practice, as current diagnostic and treatment strategies for Painful-DPN are largely empirical and do not account for clinical phenotype or disease mechanisms. These results suggest that greater attention should be given to addressing anxiety and sleep disturbances in women with Painful-DPN. Furthermore, future studies investigating new therapeutics for Painful-DPN should include subgroup analyses to evaluate efficacy in women vs men. This study opens new research avenues, particularly in exploring whether sex-specific disease mechanisms exist in Painful-DPN and whether tailored interventions for each sex could enhance treatment outcomes for this debilitating condition.^27^

Although there are strengths to this study, administering multiple PROMs, detailed neurological phenotyping, and matching of male and female cohorts, we acknowledge that there are limitations. This was a retrospective, exploratory analysis of cross-sectional data collected over 15 years with predetermined selection criteria, precluding causal inference. The study population comprised 2 separate Painful-DPN cohorts, one undergoing detailed psychometric and quality of life assessment and the other extensive neurophysiological evaluation; although standardised procedures were used, the retrospective design resulted in different diagnostic criteria and grading of certainty for Painful-DPN (probable and confirmed), which may introduce heterogeneity, and there was missing data, particularly for PROM assessments. Participants were assessed by different researchers, although all were trained in the same manner. Recruitment from cross-sectional studies introduces potential selection bias, although no remuneration was provided, and multiple comparisons and incomplete data may have reduced statistical power. Finally, the absence of participants with nonpainful-DPN and without DPN limits the ability to distinguish sex-related differences independent of neuropathic pain (eg, anxiety and sleep), and in the PROM analysis, there was a group difference in type 1 diabetes vs men, and future studies would need matching of type of diabetes.

To conclude, this study found that women with Painful-DPN have a greater burden of disease with more severe impairment of small-nerve fibre function. Large prospective studies with careful pain/neurological phenotyping with assessment of small-fibre function (eg, using skin biopsy) are urgently needed to shed light on to provide greater mechanistic insight into this prevalent condition.

## Disclosures

The authors have no conflict of interest to declare.

## Supplemental digital content

Supplemental digital content associated with this article can be found online at http://links.lww.com/PR9/A406.
