# Supplementary material for: Higher disease burden and greater small fibre impairment in women with painful diabetic neuropathy
Source: Pain Rep. 2026 Apr 28;11(3):e1443. doi: 10.1097/PR9.0000000000001443 (PMC13132318; doi:10.1097/PR9.0000000000001443)
Supplement: Supplementary file 1 [file painreports-11-e1443-s001.pdf]

Supplementary Materials

Quantitative Sensory Testing Protocol

Quantitative sensory testing (QST) was performed according to the German Research Network on Neuropathic Pain (DFNS) protocol.(1) Cold and warm detection thresholds (CDT and WDT), as well as cold and heat pain thresholds (CPT and HPT), thermal sensory limens (TSL) and paradoxical heat sensations (PHS), were established using a MEDOC TSA-II Neurosensory Analyser (Ramat Yishai, Israel). Also assessed were, mechanical detection (MDT) and pain thresholds (MPT) and mechanical pain sensitivity (MPS), allodynia, pressure pain thresholds (PPT), wind-up ratio (WUR) and vibration detection thresholds. MDT was assessed with a set of standardised von Frey filaments (0.25, 0.5, 1, 2, 4, 8, 16, 32, 64, 128 and 256 mN; Nervtest, Marstock, Germany) using a modified method of limits. MPT was assessed with a set of seven metal probes of standardised stimulus intensities (8, 16, 32, 64, 128, 256 and 512 mN; MRC Systems – Medizintechnische Systeme, Heidelberg, Germany), using a uniform skin contact area of 0.25 mm and a modified method of limits. MPS and DMA were determined using the same set of seven metal probes with standardised stimulus intensities and, in addition, a set of seven light intensity stimuli: a cotton wool ball with a force of 3 mN; a Q-tip (fixed to a plastic stick) with a force of 100 mN; and a paintbrush with an applied force of 200–400 mN. These stimuli were applied 50 times (five runs of ten stimuli per test site in different pseudo-randomised sequence), and the participants were asked to rate the intensity of each stimulus on a 0–100 numeric rating scale (0, no pain; 100, most severe pain). The WUR, as a measure of enhanced temporal summation, was examined by a pinprick stimulus of standardised intensity (256 mN). The stimulus was first applied singularly and then in a series of ten stimuli with a frequency of 1 Hz within an area of 1 cm^2 with participants asked to rate the intensity of the first stimulus and the mean of ten stimuli on a scale of 0–100. The ratio between the two measures was calculated as WUR; a WUR of >1 indicates enhanced temporal

summation. The vibration detection threshold was examined using a tuning fork (64 Hz, 8/8 scale) at the (lateral or medial) malleolus area. Muscular pressure pain threshold was examined by applying mechanical pressure at a rate of 0.5 kg/s (Algometer, Somedic, Sweden) at the abductor halluces muscle. Except for the vibration detection threshold and pressure pain threshold, all sensory tests were performed in the S1 dermatome bilaterally (unless defined by the distribution of symptoms). PPTs were recorded over the arch of the foot and vibration detection thresholds were tested over the medial malleolus. The QST data were entered into the data analysis system eQUISTA provided by the DFNS. eQUISTA transformed the raw QST data into *z* scores thus normalising for age, sex and the body location of testing.(2) Positive *z* scores denote gain of function, whereas negative *z* scores denote loss of function.

Results

	Females (n=56)	Males (n=96)	p-value
Intensity	7.0 (3.0)	7.0 (3.0)	0.106
Sharp	8.0 (4.0)	7.0 (4.0)	0.559
Hot	7.0 (6.0)	6.0 (6.0)	0.055
Dull	6.0 (4.0)	5.0 (5.0)	0.109
Cold	2.0 (8.0)	0.0 (4.8)	0.194
Sensitive	6.0 (7.0)	4.5 (8.0)	0.240
Itch	2.0 (7.0)	0.0 (4.0)	**0.034**
Unpleasant	8.0 (3.0)	8.0 (4.0)	0.366
Deep	8.0 (3.0)	7.0 (3.8)	0.123
Surface	5.0 (3.0)	5.5 (4.0)	0.870
Total Score	56.3 ± 18.3	49.8 ± 19.3	**0.021**†

Table 1. Neuropathic Pain Scale results of participants undergoing analysis of Patient Reported Outcome Measures grouped by sex. Data are presented as mean (±SD) or median (IQR). Boldface text denotes significant results. Tests were Mann-Whitney U, unless otherwise stated: † independent t-test.

	Females (58)	Males (91)	p-value
Pain Catastrophizing scale			
Rumination	6.0 (9.0)	7.0 (9.0)	0.819
Magnification	3.0 (5.0)	2.0 (5.0)	0.536
Helplessness	8.0 (13.0)	6.0 (11.0)	0.332
Total Score	18.0 (24.0)	13.0 (22.0)	0.547
Chronic Pain Acceptance Questionnaire			
Activities Engagement	36.0 (17.0)	37.5 (26.0)	0.646
Pain Willingness	28.0 (20.0)	28.0 (24.0)	0.584

Table 2. Pain Catastrophizing scale and Chronic Pain Acceptance Questionnaire results of participants undergoing analysis of Patient Reported Outcome Measures grouped by sex. Data are presented as median (IQR) and the test was Mann-Whitney U.

	Females<br><br>(Spearman's r, p-value)	Males<br><br>(Spearman's r, p-value)
NIS-LL+7	0.128, 0.492	**0.360, 0.008**
CDT, *z*-score	-0.254, 0.175	-0.061, 0.696
WDT, *z*-score	-0.191, 0.312	0.236, 0.128
TSL, *z*-score	-0.343, 0.063	0.199, 0.202
CPT, *z*-score	-0.131, 0.490	0.087, 0.578
HPT, *z*-score	-0.184, 0.331	0.273, 0.077
PPT, *z*-score	**-0.427, 0.018**	-0.016, 0.917
VDT, *z*-score	0.034, 0.857	**-0.460, 0.002**
MPT, *z*-score	-0.053, 0.797	-0.108, 0.514
MPS, *z*-score	-0.166, 0.418	-0.100, 0.545
WUR, *z*-score	0.693, 0.057	0.339, 0.257
MDT, *z*-score	-0.281, 0.165	**-0.526, <0.001**

Table 3. Spearman's Correlation Analysis between 24-hour Numeric Rating Scale for neuropathic pain intensity and neurophysiological and DFNS QST measures. Boldface text denotes significant results. NIS-LL, neuropathy impairment score of the lower limbs; TCNS, Toronto clinical scoring system; NIS-LL+7, neuropathy impairment score of the lower limbs plus seven neurophysiological tests; CDT, cold detection threshold; WDT, warm detection threshold; TSL, thermal sensory limen; CPT, cold pain threshold; HPT, heat pain threshold; PPT, pressure pain threshold; VDT, vibration detection threshold; MPT, mechanical pain threshold; MPS, mechanical pain sensitivity; MDT, mechanical detection threshold; WUR, wind-up ratio

1. Rolke R, Baron R, Maier C, Tölle TR, Treede RD, Beyer A, et al. Quantitative sensory testing in the German Research Network on Neuropathic Pain (DFNS): standardized protocol and reference values. Pain. 2006;123(3):231-43.
2. Magerl W, Krumova EK, Baron R, Tölle T, Treede RD, Maier C. Reference data for quantitative sensory testing (QST): refined stratification for age and a novel method for statistical comparison of group data. Pain. 2010;151(3):598-605.
